# A Single-Center Study on the Outcomes of Target Limb Revascularization in Femoropopliteal Lesions Treated With Drug Coated Balloons and Bare Metal Stents

**DOI:** 10.1177/15266028211068772

**Published:** 2022-01-05

**Authors:** Victoria Linehan, Maria Doyle, Brendan Barrett, Ravindra Gullipalli

**Affiliations:** 1 Faculty of Medicine, Memorial University of Newfoundland, St. John’s, NL, Canada; 2 Discipline of Radiology, St. Clare’s Mercy Hospital, St. John’s, NL, Canada

**Keywords:** femoropopliteal disease, endovascular treatment, bare metal stents, drug coated balloons, revascularization

## Abstract

**Purpose:**

Multiple randomized controlled trials have shown that both drug coated balloons (DCBs) and bare metal stents (BMSs) significantly reduce restenosis in femoropopliteal lesions compared with plain balloon angioplasty. However, few studies have directly compared DCB and BMS treatments. Therefore, the goal of our study was to determine if the rate of target lesion revascularization (TLR) differs between DCB and BMS treatment at our center.

**Materials and methods:**

We performed a retrospective chart review of femoropopliteal interventions at a single center from 2009 to 2017. The intervention, patient and lesion characteristics, and TLR events were recorded. Exclusion criteria were loss of follow-up, death, bail-out stenting, and amputation within 60 days of treatment. Freedom from TLR was analyzed over a 3 year period with Kaplan-Meier survival curves. Cox hazard ratios were calculated to account for patient and lesion characteristics.

**Results:**

A total of 322 lesions (234 patients) treated with DCBs and 225 lesions (194 patients) treated with BMSs were included in this study. There were significant differences in baseline patient and lesion characteristics between groups—a greater proportion of women, patients with dyslipidemia, and lesions with popliteal involvement were treated with DCBs. There was no difference in the freedom from TLR between DCBs and BMSs. Accounting for patient and lesion characteristics, there was still no difference between DCBs and BMSs on the hazard of TLR. While our analysis did not detect a difference in the rate of TLR, there was a significant difference in the type of TLR. Compared with DCBs, a greater proportion of lesions initially treated with BMSs were retreated via surgical bypass rather than endovascular intervention, suggesting that lesions treated with DCBs may be more amenable to future endovascular intervention.

**Conclusion:**

Our retrospective analysis showed no difference in the rate of TLR between lesions treated with DCBs and BMSs. However, DCBs were more often used in complicated lesions involving popliteal arteries and may also allow for easier endovascular reintervention.

## Introduction

The treatment of atherosclerosis in femoropopliteal arteries poses a significant challenge due to high rates of lesion recurrence^1–3^ and significant patient morbidity.^4^ While endovascular treatment was initially limited to plain balloon angioplasty (PBA), bare metal stents (BMSs) and paclitaxel drug coated balloons (DCBs) have since both been shown to improve patient outcomes and decrease lesion recurrence.^5–11^ Few studies directly compare BMSs against DCBs, and despite insufficient evidence to support improved clinical outcomes, DCBs have been most often chosen in clinical practice.^12^ Recently, a meta-analysis showed that paclitaxel devices, including DCBs, increased the risk of death at 2 and 5 years post intervention.^13^ This has made clinicians reconsider the role of DCBs in clinical practice. While evidence is accumulating to address the safety of DCBs,^14–16^ research is required to compare the efficacy of DCBs against potentially safer alternatives like BMSs to develop an appropriate risk-benefit assessment in femoropopliteal disease management. Therefore, the goal of our study was to retrospectively compare lesions treated with BMSs or DCBs at our center to determine which was most effective to limit lesion recurrence.

## Materials and Methods

We performed a single-center retrospective chart review of all femoropopliteal lesions treated with PBA, BMSs, paclitaxel DCBs, and paclitaxel drug-eluting stents (DESs) between December 2009 and December 2017 in the Eastern Health region of Newfoundland and Labrador, Canada. This dataset was analyzed in a previous study on the risk of mortality in our patient population.^17^ For the purposes of this article, we used a subset of these data to compare lesions treated with BMSs and DCBs, excluding lesions treated with PBA and DESs. Exclusion criteria were patients who died during the follow-up period, patients lost to follow-up, patients who had the respective limb amputated within 60 days (revascularization was likely performed for post-op healing), lesions treated with DCBs that required bail-out stenting, and lesions with missing data.

We collected data on patient demographics (age and sex), patient risk factors, lesion characteristics, the presence of critical limb ischemia as defined by the Rutherford classification, and TransAtlantic Inter-Society Consensus for the Management of Peripheral Arterial Disease (TASC II) level. Risks factors were smoking defined by patient/physician reported history, diabetes defined as a hemoglobin A1C above 6.5%, dyslipidemia based on LDL greater than 3.5 mmol/L or the use of a statin, hypertension based on antihypertensive use or established diagnosis, and chronic kidney disease defined by an eGFR of less than 60 for at least 1 year. Lesion characteristics included if they were multifocal (iliac arteries, superficial femoral artery, popliteal arteries, or infra-popliteal vessels), if there was an occlusion versus stenosis, and lesion length.

Patients were followed up for 3 years post-intervention to record primary and secondary outcomes. Primary outcomes were termed target lesion revascularization (TLR) and classified as repeat endovascular intervention or surgical bypass, while secondary outcomes were major lower limb amputations and death.

Patient and lesion characteristics were analyzed with 1- or 2-tailed chi-square tests and *t*-tests, as appropriate. Freedom from TLR was analyzed using a Kaplan-Meier survival curve with a log-rank test to compare BMSs and DCBs. To control for patient and lesion characteristics, a Cox regression was performed to calculate hazard ratios (HRs) for each variable. A backward stepwise variable selection method using the likelihood ratio test was used to refine our model. Schoenfeld residuals were used to ensure hazards were proportional over time to satisfy assumptions of this refined model. Data were analyzed with GraphPad Prism and R software. A p<0.05 was considered significant.

This study was approved by the Newfoundland and Labrador Health Research Ethics Board with permission from our regional health authority’s Research Proposals Approval Committee. This study did not require informed consent. There were no sources of funding or conflicts of interest for this project.

## Results

**Figure 1.**
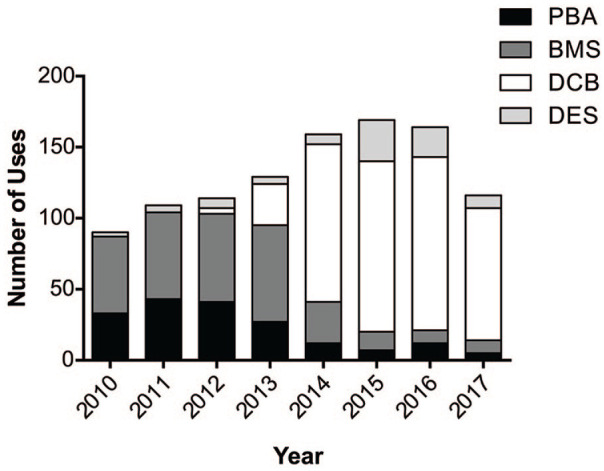
Trends of endovascular treatment at our center from 2010 to 2018. PBA, plain balloon angioplasty; BMS, bare metal stent; DCB, drug coated balloon; DES, drug-eluting stent.

Our chart review identified 1054 lesions from 702 patients who were treated endovascularly between December 2009 and December 2017. As DCBs were first introduced at our center in late 2012, we first plotted the number of lesions treated with PBA, BMSs, DCBs, or DESs to determine the impact on our clinical practice (Figure 1). While BMS was the preferred endovascular treatment at our center between 2010 and 2013, DCB was mainly used from 2014 onward, treating 69.81% to 80.17% of lesions each year.

**Figure 2.**
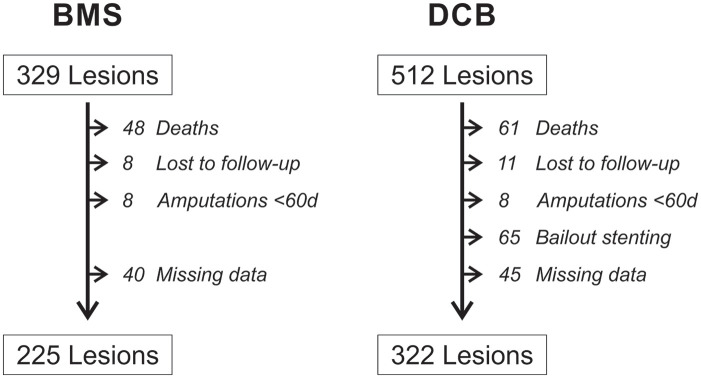
Flow chart of lesion selection and exclusion criteria. BMS, bare metal stent; DCB, drug coated balloon.

To assess recurrence rates between these 2 most common endovascular treatments, we extracted a subset of our data to compare 329 lesions treated with BMSs versus 512 lesions treated with DCBs. After applying our exclusion criteria, a total of 225 lesions (194 patients) treated with BMSs and 322 lesions (234 patients) treated with DCBs were included in our analysis (Figure 2).

**Figure 3.**
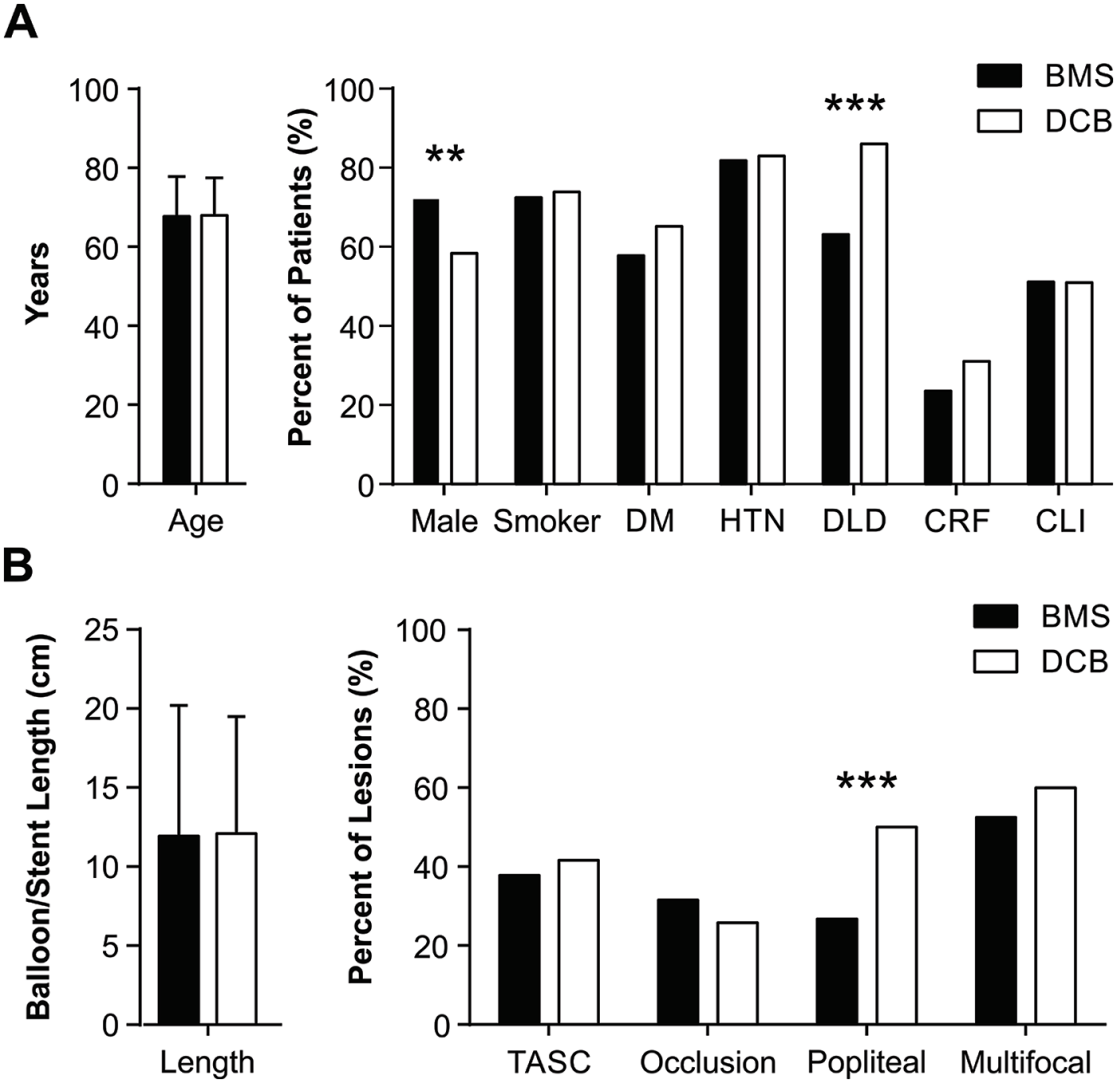
Pre-existing differences in baseline patient (A) and lesion (B) characteristics between bare metal stent (BMS, black) and drug coated balloon (DCB, white) groups. DM, diabetes mellitus; HTN, hypertension; DLD, dyslipidemia; CRF, chronic renal failure; CLI, critical limb ischemia. **p<0.01. ***p<0.001.

Initially, we compared baseline characteristics between the BMS and DCB groups (Figure 3). While patient age was similar between groups, a higher proportion of women were treated with DCBs (χ^2^=10.65, df=1, p=0.001; Figure 3A). Most vascular disease risk factors were similar between groups except that significantly more patients with dyslipidemia were treated with DCBs (χ^2^=38.80, df=1, p≤0.001; Figure 3A). The percentage of patients with critical limb ischemia was similar between groups but notably this percent was very high in our patient population: 51.11% for BMSs and 50.93% for DCBs. In terms of lesion characteristics, there was no difference in TASC level, the lesion length, presence of occlusion, or multifocal disease. However, a higher proportion of patients with popliteal involvement were treated with DCBs (BMSs: 26.67% vs DCBs: 50.00% popliteal lesions; χ^2^=29.95, df=1, p≤0.001; Figure 3B).

**Figure 4.**
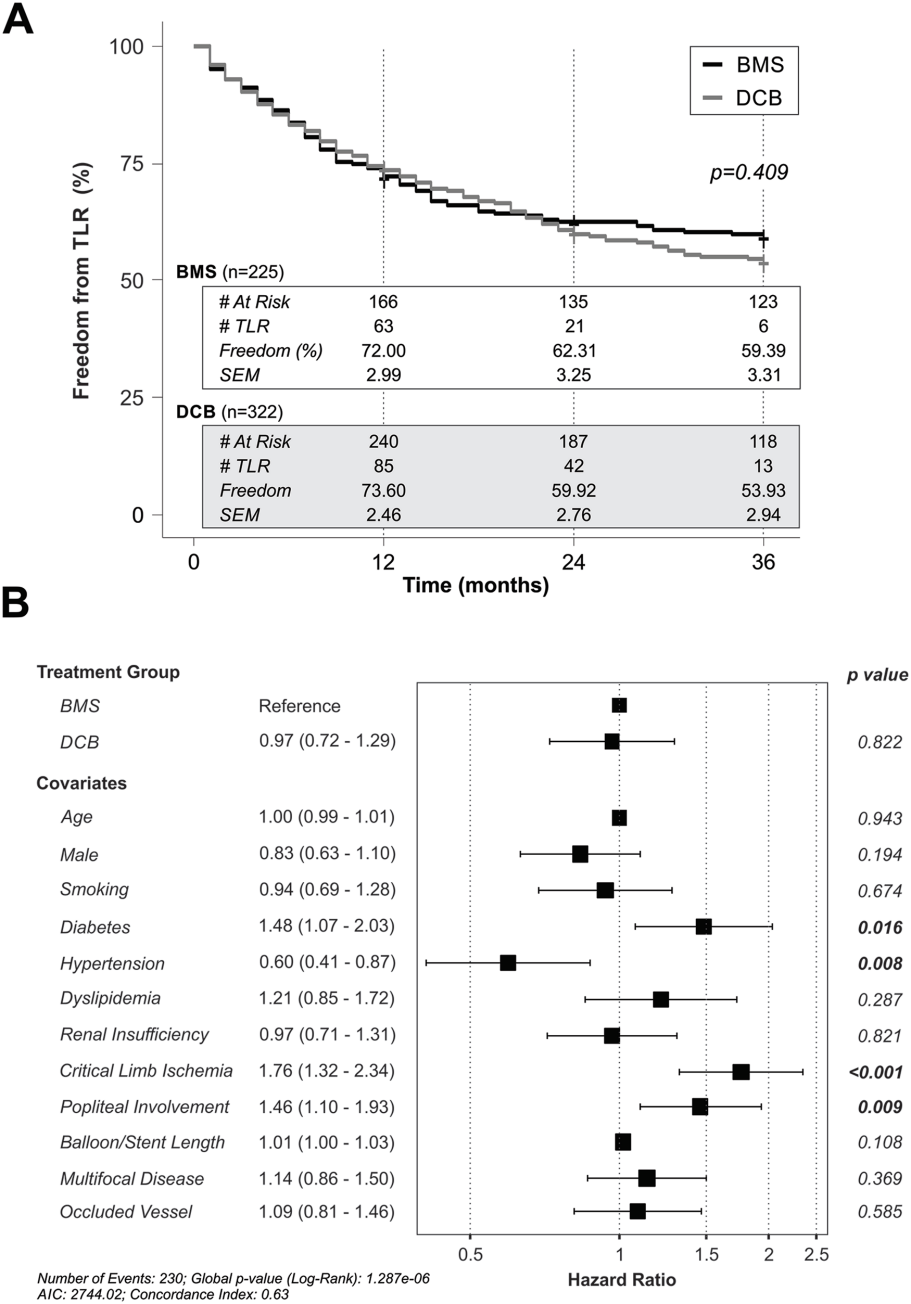
Rates of target lesion revascularization (TLR) over a 3 year period at our center. (A) Kaplan-Meier analysis of freedom from TLR in lesions treated with BMSs (bare metal stents, black) or DCBs (drug coated balloons, gray). (B) Cox regression analysis of the hazard of TLR with all covariates included.

**Figure 5.**
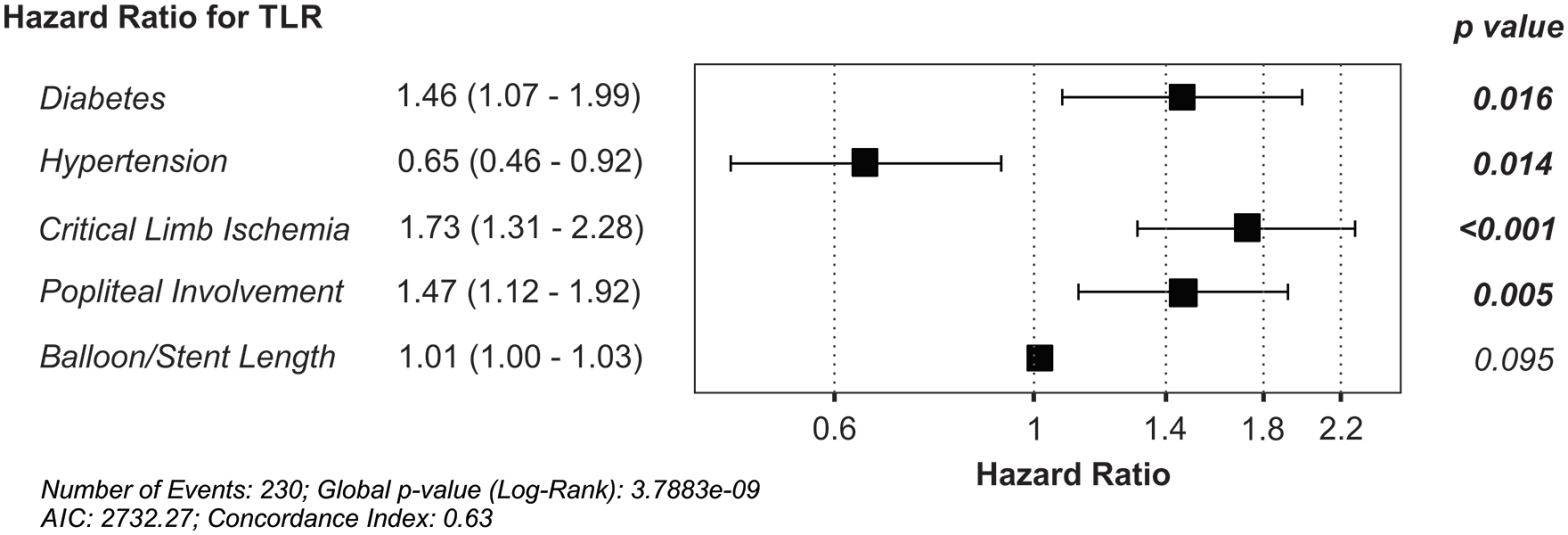
Final model for the hazard of target lesion revascularization (TLR) over a 3 year period at our center. AIC, Akaike information criterion.

To compare the rates of TLR between groups, we performed a Kaplan-Meier survival analysis on the recurrence of lesions requiring retreatment over a 3 year period. There was no difference in TLR over time between BMS and DCB treatments (χ^2^=0.68, df=1, p=0.409; Figure 4). As there were pre-existing differences between groups, we next performed a Cox regression analysis to control for confounding variables. In our first iteration, we found that the HR for DCBs did not differ from BMSs, again demonstrating that there is no difference in recurrence rates between these 2 treatments (Figure 4). However, the presence of diabetes, critical limb ischemia, popliteal involvement, and hypertension was significantly associated with TLR. To further refine our hazard model, we performed a backward stepwise selection of variables to identify the main contributory factors to TLR. Our final model (Figure 5) was reduced to only 5 variables. The presence of critical limb ischemia (HR, 1.73 [1.31–2.28], p<0.001), popliteal involvement (HR, 1.47 [1.12–1.92], p=0.005), and diabetes (HR, 1.46 [1.07–1.99], p=0.016) was a significant predictor of TLR, with the length of the lesion showing only a small hazard with a trend to predict TLR (HR, 1.01 [1.00–1.03], p=0.095). In contrast, the presence of hypertension was associated with a reduced hazard of TLR (HR, 0.65[0.46–0.92], p=0.014).

**Figure 6.**
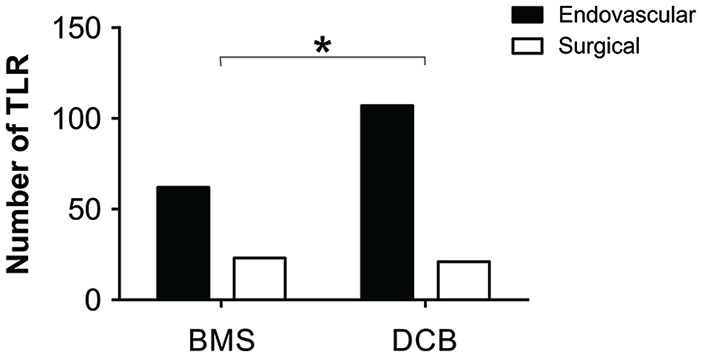
Proportion of primary TLR of either endovascular retreatment (black) or surgical bypass (white) in lesions initially treated with BMSs or DCBs. TLR, target lesion revascularization; BMS, bare metal stent; DCB, drug coated balloon. *p<0.05.

Although there was no difference in the TLR between BMSs and DCBs, we next assessed the other clinical considerations between the 2 treatment modalities. Most TLR were primary, either repeat endovascular treatment or surgical bypass, but there were a few secondary TLR (5 amputations BMSs and 12 amputations in DCBs). As in-stent stenosis is difficult to revascularize,^18^ we hypothesized that there may be less BMS-treated lesions that are amenable to repeat endovascular treatment. To test this, we compared the proportion of primary TLR between groups and found that a higher proportion of lesions initially treated with DCBs underwent repeat endovascular intervention compared with BMSs (χ^2^=3.54, df=1, p=0.030; Figure 6). When assessing these recurrent lesions, we also found that DCBs are chosen to treat 79.90% of recurrent lesions, a significant difference compared with BMSs (χ^2^=69.36, df=1, p≤0.001). Finally, while DCBs may be the preferred treatment for de novo and recurrent lesions, they are also associated with revascularization failure requiring bail-out stenting with either BMS (60.00%) or DES (40.00%). Bail-out stenting was not uncommon in our dataset, and the rates were comparable in de novo (13.17%) and recurrent (11.61%) lesions.

## Discussion

In this study, we found that the rate of TLR does not differ between femoropopliteal lesions treated with DCBs or BMSs. Rather, critical limb ischemia, diabetes, and popliteal involvement are associated with a higher risk of TLR, while hypertension is associated with a lower risk of TLR. Although it may be counterintuitive that hypertension has a protective effect in treated femoropopliteal lesions, it is likely that the reduction of TLR in this group is due to antihypertensive treatment rather than the presence of hypertension itself.

Multiple randomized controlled trials, retrospective studies, and meta-analyses have shown decreased lesion recurrence and improved clinical outcomes in patients treated with BMSs compared with PBA,^5,6^ and with DCBs compared with PBA.^5,7–11^ In keeping with this evidence, these treatments were the main endovascular interventions for femoropopliteal disease at our center, with a shift to significantly favor DCBs after its introduction in 2012. A similar practice trend has been observed nationally in the United States as DCB is the most popular endovascular treatment of femoropopliteal lesions.^12^ Despite the general preference for DCBs, few studies have compared the efficacy between BMSs and DCBs to prevent lesion recurrence. Although no randomized control trials exist to directly compare BMSs with DCBs, a network meta-analysis found a modestly lower rate of TLR with DCB treatment when indirectly compared with BMSs,^5^ while another indirect meta-analysis showed no difference in TLR rate.^19^ Similarly, our single-center study found no difference in the rate of TLR in lesions treated with BMSs or DCBs. In current literature, there have been only 2 other studies that directly compared BMSs and DCBs. Both were retrospective analyses with the first showing no difference in the rate of TLR, albeit with a small sample size,^20^ and the second, a propensity-matched analysis, showing that DCBs had decreased patency compared with nitinol BMSs.^21^ While our data appear to be conflict with the latter, it is important to consider that both were retrospective cohort studies and so the assignment of lesions to DCB or BMS treatment groups was not random but instead a result of clinician judgment and decision-making that will differ between centers.

Focusing on the results of our study, we cannot definitively conclude that DCBs and BMSs have equivalent outcomes without a controlled study design. It is possible that our clinicians carefully selected which intervention was best suited for each lesion based on their experience to optimize patient outcomes. For example, if a specific intervention were the preferred approach for difficult-to-treat lesions that would have a high rate of TLR regardless of treatment modality, this may falsely elevate the incidence of TLR for that intervention. While we performed a multivariate regression to correct for such differences, statistical analyses cannot anticipate all contributing and interacting factors. Biased lesion selection could explain why there were significant pre-existing differences in the characteristics of patients and lesions. For example, DCB was more often chosen for lesions that have popliteal involvement at our center, which was found to be an independent risk factor for TLR in our study and others.^22,23^

An important consideration in the treatment of femoropopliteal disease is the technical challenge of reintervention for lesions first treated with BMSs compared with DCBs. In-stent stenosis is known to have lower patency and higher TLR than restenosis.^22,24^ This difficulty to reintervene for in-stent stenosis may be reflected in our study as the proportion of recurrent lesions that were treated with bypass was significantly greater in the BMS group compared with the DCB group. In contrast, restenosis of lesions treated with DCBs has shorter lesion lengths and is easier to treat.^24^ DCBs are more often chosen to treat in-stent stenosis at our center, as overlapping stents increase both the rate of TLR and the risk of stent fracture.^25^ Even so, DCB treatment of in-stent stenosis still has low patency when compared against de novo and restenosis.^24^

Finally, although not a factor for decision making during our study period, clinicians may now be wary of using paclitaxel DCBs in light of a recent meta-analysis that suggested an increased risk of mortality at 2 and 5 years post-paclitaxel treatment.^13^ However, our patient population does not show evidence of increased mortality.^17^ Moreover, large studies that include patient-level data do not find an association with drug coated devices and death.^14–16^

To summarize, the decision to treat femoropopliteal lesions either with BMSs or DCBs is complex. Research is limited to indirect comparisons from meta-analyses and limited retrospective studies that introduce bias through each interventionalist’s clinical judgment. Through our own experience and in line with evidence in the literature,^25^ we would recommend using DCBs as the first-line treatment as a high rate of recurrence is expected in femoropopliteal disease, and it is technically easier to treat restenosis than treating in-stent stenosis. We also prefer DCBs for multifocal lesions that span a large area and lesions involving the popliteal artery. We consider BMS to be a second-line treatment reserved for complicated or heavily calcified lesions.^24,26^ Although we advocate for initial treatment with DCBs, it should be recognized that DCBs have a notable rate of intraprocedural failure requiring bail-out stenting, 13.17% at our center which is comparable with other reports.^20,27^ In addition, there remains a question about increased mortality risk with paclitaxel DCBs, although we have no evidence to support this in our patient population.^17^

There were several limitations to our study. One limitation was the retrospective nature of the study in which clinical judgment may have introduced selection bias into treatment groups as discussed above. In keeping with this limitation, there were pre-existing differences in our groups and possible unknown variables that may have confounded our results. A further systematic issue with the retrospective data collection is that due to the change in treatment choice over time, lesions were more often treated with BMSs earlier in the study period with most lesions being treated 4 to 5 years before DCBs. This required us to start data collection for BMSs 3 years prior to the first use of DCBs at our center to have a sufficient sample size for our analysis. While there has been changes in the natural history of lower extremity peripheral arterial disease since the 1980s, such comparisons have been made on the order of decades and precede the period of our study.^28^ Therefore, it is unclear whether time of treatment affected our results. Finally, our patient population has a high rate of comorbidity with around half of the patients presenting with critical limb ischemia. This may limit the applicability of our results to healthier populations.

Future directions for our research would be to do a longitudinal analysis of de novo lesions treated with BMSs or DCBs to assess any difference in future recurrences and revascularization. If a difference is found, this research could help develop an evidence-based long-term treatment plan for femoropopliteal disease while anticipating recurrences and preparing for repeated treatments. Such a plan would also require analysis of lesion and patient characteristics to determine prognostic factors related to each treatment, which would best be assessed by a randomized design.

In conclusion, despite the prevalent use of DCBs over other endovascular therapies, our study shows no difference in TLR between DCBs and BMSs; however, DCBs were more often used in complicated lesions, such as those involved in popliteal arteries. Therefore, to develop an evidence-based algorithm on the endovascular approach to femoropopliteal management, additional factors must be evaluated such as efficacy of treatment based on lesion characteristics and the long-term outcomes of recurrent reintervention.

## References

[1] Schillinger M Haumer M Schlerka G , et al. Restenosis after percutaneous transluminal angioplasty in the femoropopliteal segment: the role of inflammation. J Endovasc Ther. 2001;8(5):477–483. doi:10.1177/152660280100800509. 11718406

[2] Schillinger M Exner M Mlekusch W , et al. Restenosis after femoropopliteal PTA and elective stent implantation: predictive value of monocyte counts. J Endovasc Ther. 2003;10(3):557–565. doi:10.1177/152660280301000322. 12932168

[3] Ahmadi R Schillinger M Maca T , et al. Femoropopliteal arteries: immediate and long-term results with a dacron-covered stent-graft. Radiology. 2002;223(2):345–350. doi:10.1148/radiol.2232010971. 11997536

[4] Ouriel K. Peripheral arterial disease. Lancet. 2001;358(9289):1257–1264. doi:10.1016/S0140-6736(01)06351-6. 11675083

[5] Jaff MR Nelson T Ferko N , et al. Endovascular interventions for femoropopliteal peripheral artery disease: a network meta-analysis of current technologies. J Vasc Interv Radiol. 2017;28(12):1617–1627. doi:10.1016/j.jvir.2017.08.003. 29031986

[6] Laird JR Katzen BT Scheinert D , et al. Nitinol stent implantation versus balloon angioplasty for lesions in the superficial femoral artery and proximal popliteal artery. Circ Cardiovasc Interv. 2010;3(3):267–276. doi:10.1161/CIRCINTERVENTIONS.109.903468. 20484101

[7] Rosenfield K Jaff MR White CJ , et al. Trial of a paclitaxel-coated balloon for femoropopliteal artery disease. N Engl J Med. 2015;373(2):145–153. doi:10.1056/NEJMoa1406235. 26106946

[8] Tepe G Zeller T Albrecht T , et al. Local delivery of paclitaxel to inhibit restenosis during angioplasty of the leg. N Engl J Med. 2008;358(7):689–699. doi:10.1056/NEJMoa0706356. 18272892

[9] Dake MD Ansel GM Jaff MR , et al. Durable clinical effectiveness with paclitaxel-eluting stents in the femoropopliteal artery. Circulation. 2016;133(15):1472–1483. doi:10.1161/CIRCULATIONAHA.115.016900. 26969758 PMC4823823

[10] Bausback Y Willfort-Ehringer A Sievert H , et al. Six-month results from the initial randomized study of the Ranger paclitaxel-coated balloon in the femoropopliteal segment. J Endovasc Ther. 2017;24(4):459–467. doi:10.1177/1526602817710770. 28558502

[11] Laird JR Schneider PA Tepe G , et al. Durability of treatment effect using a drug coated balloon for femoropopliteal lesions. J Am Coll Cardiol. 2015;66(21):2329–2338. doi:10.1016/j.jacc.2015.09.063. 26476467

[12] Mohapatra A Saadeddin Z Bertges DJ , et al. Nationwide trends in drug coated balloon and drug-eluting stent utilization in the femoropopliteal arteries. J Vasc Surg. 2020;71(2):560–566. doi:10.1016/j.jvs.2019.05.034. 31405761 PMC7007839

[13] Katsanos K Spiliopoulos S Kitrou P , et al. Risk of death following application of paclitaxel-coated balloons and stents in the femoropopliteal artery of the leg: a systematic review and meta-analysis of randomized controlled trials. J Am Heart Assoc. 2018;7(24):e011245. doi:10.1161/JAHA.118.011245. PMC6405619 30561254

[14] Secemsky EA Kundi H Weinberg I , et al. Association of survival with femoropopliteal artery revascularization with drug coated devices. JAMA Cardiol. 2019;4(4):332–340. doi:10.1001/jamacardio.2019.0325. 30747949 PMC6484791

[15] Freisinger E Koeppe J Gerss J , et al. Mortality after use of paclitaxel-based devices in peripheral arteries: a real-world safety analysis. Eur Heart J. 2020;41:3732–3739. doi:10.1093/eurheartj/ehz698. 31593987 PMC7666867

[16] Schneider PA Laird JR Doros G , et al. Mortality not correlated with paclitaxel exposure: an independent patient-level meta-analysis of a drug coated balloon. J Am Coll Cardiol. 2019;73(20):2550–2563. doi:10.1016/j.jacc.2019.01.013. 30690141

[17] Linehan V Doyle M Barrett B , et al. A single-center experience of paclitaxel in the treatment of femoropopliteal disease—no evidence for an association with mortality. Vasc Endovascular Surg. 2020;54(5):400–405. doi:10.1177/1538574420920994. 32319355

[18] Rymer JA Jones WS. Femoropopliteal in-stent restenosis. Circ Cardiovasc Interv. 2018;11(12):e007559. doi:10.1161/CIRCINTERVENTIONS.118.007559. 30562092

[19] Fusaro M Cassese S Ndrepepa G , et al. Paclitaxel-coated balloon or primary bare nitinol stent for revascularization of femoropopliteal artery: a meta-analysis of randomized trials versus uncoated balloon and an adjusted indirect comparison. Int J Cardiol. 2013;168(4):4002–4009. doi:10.1016/j.ijcard.2013.06.081. 23890909

[20] Allam AK Ismail M. Drug-eluting balloon angioplasty versus bare-metal stent in treating chronic total occlusion of femoropopliteal arterial segment: a review of 1-year outcome of 90 patients with TASC C and D lesions. Egypt J Surg. 2019;38:204–213. doi:10.4103/ejs.ejs_146_18.

[21] Steiner S Schmidt A Bausback Y , et al. Midterm patency after femoropopliteal interventions. J Endovasc Ther. 2016;23(2):347–355. doi:10.1177/1526602816628285. 26848131

[22] Micari A Brodmann M Keirse K , et al. Drug coated balloon treatment of femoropopliteal lesions for patients with intermittent claudication and ischemic rest pain. JACC Cardiovasc Interv. 2018;11(10):945–953. doi:10.1016/j.jcin.2018.02.019. 29798770

[23] Stavroulakis K Torsello G Manal A , et al. Results of primary stent therapy for femoropopliteal peripheral arterial disease at 7 years. J Vasc Surg. 2016;64(6):1696–1702. doi:10.1016/j.jvs.2016.05.073. 27575816

[24] Schmidt A Piorkowski M Görner H , et al. Drug coated balloons for complex femoropopliteal lesions. JACC Cardiovasc Interv. 2016;9(7):715–724. doi:10.1016/j.jcin.2015.12.267. 27056311

[25] AbuRahma AF. When are endovascular and open bypass treatments preferred for femoropopliteal occlusive disease? Ann Vasc Dis. 2018;11(1):25–40. doi:10.3400/avd.ra.18-00001. 29682105 PMC5882358

[26] Fanelli F Cannavale A Gazzetti M , et al. Calcium burden assessment and impact on drug-eluting balloons in peripheral arterial disease. Cardiovasc Intervent Radiol. 2014;37(4):898–907. doi:10.1007/s00270-014-0904-3. 24806955

[27] Zeller T Rastan A Macharzina R , et al. Drug coated balloons vs. drug-eluting stents for treatment of long femoropopliteal lesions. J Endovasc Ther. 2014;21(3):359–368. doi:10.1583/13-4630MR.1. 24915582

[28] Teraa M Conte MS Moll FL , et al. Critical limb ischemia: current trends and future directions. J Am Heart Assoc. 2016;5(2):e002938. doi:10.1161/JAHA.115.002938. PMC4802465 26908409

